# Supplementary material for: Analyzing Trajectories of Acute Cigarette Reduction Post-Introduction of an E-Cigarette Using Ecological Momentary Assessment Data
Source: Int J Environ Res Public Health. 2022 Jun 17;19(12):7452. doi: 10.3390/ijerph19127452 (PMC9223631; doi:10.3390/ijerph19127452)

Supplementary Tables and Figures

Supplementary Figure S1. CONSORT diagram of analytical sample

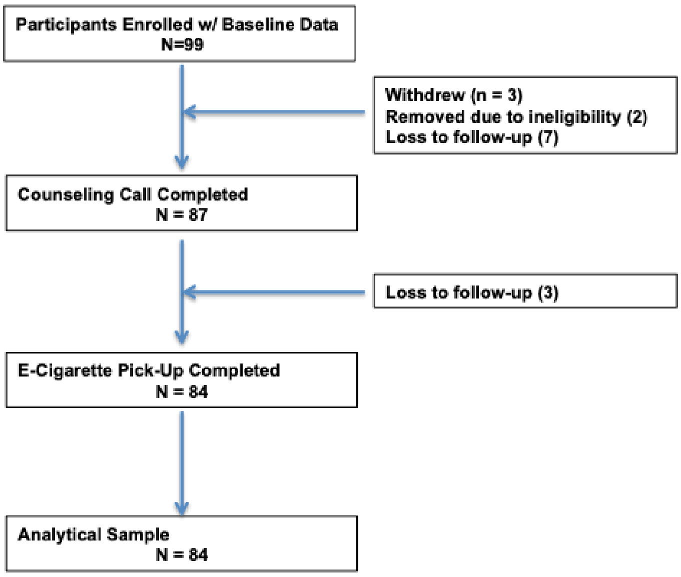

Supplementary Table S1. Model fit indices for trajectory determination, assessed via Nagin Clustering

Group	BIC	AIC	Log Likelihood
2-class model	-2498.76	-2489.04	-2481.04
3-class model	-2376.46	-2364.30	-2354.30
4-class model	-2356.93	-2342.35	-2330.35
5-class model	-2347.90	-2328.45	-2312.45

Supplementary Table S2. Unadjusted Associated Factors for Trajectory Group Assignment in Multinomial Logistic Regression Model: with Relative Risk Ratio (Reference: Maintainers)

	Relative Risk Ratio (95% CI)		
	**Rapid Reducers**	**Moderate Reducers**	**Slow Reducers**
Demographics			
Age in years	1.00 (0.85-1.18)	0.99 (0.83-1.18)	0.99 (0.84-1.16)
Gender			
Male (ref)	1.0 (Ref)	1.0 (Ref)	1.0 (Ref)
Female	1.3 (0.35-4.68)	8.0 (1.4-46.8)	2.9 (0.78-10.53)
Education			
High school or less (ref)	1.0 (Ref)	1.0 (Ref)	1.0 (Ref)
Some college	0.83 (0.15-4.64)	1.25 (0.21-7.62)	0.50 (0.09-2.84)
College/Post-Graduate	0.50 (0.09-2.84)	0.43 (0.06-2.97)	0.80 (0.16-4.08)
Race/Ethnicity			
Non-Hispanic African American/Black (Ref)	0.60 (0.12-2.97)	2.4 (0.39-14.9)	0.53 (0.10-2.84)
Non-Hispanic white	1.0 (Ref)	1.0 (Ref)	1.0 (Ref)
Other non-Hispanic	1.2 (0.09-16.24)	6.0 (0.42-85.25)	5.3 (0.52-54.34)
Hispanic of any race	0.75 (0.14-3.94)	3.0 (0.46-19.59)	1.0 (0.20-5.12)
Treatment			
Control (Placebo)	1.0 (Ref)	1.0 (Ref)	1.0 (Ref)
Active Nicotine	1.09 (0.30-3.91)	0.8 (0.21-3.09)	1.08 (0.31-3.71)
Tobacco Use			
Baseline Cigarettes Per Day			
10	1.0 (Ref)	1.0 (Ref)	1.0 (Ref)
> 10	0.15 (0.02-1.39)	0.13 (0.01-1.26)	0.16 (0.02-1.41)
Heaviness of Smoking Index (0-5 scale)	0.58 (0.32-1.05)	0.70 (0.38-1.28)	0.92 (0.52-1.62)
Made serious quit attempts (>1 day) in last year	2.6 (0.70-9.64)	1.7 (0.42-6.56)	1.3 (0.38-4.72)
Confidence in Quit Ability (0-10)	1.29 (1.0-1.67)^	1.39 (1.05-1.85)*	1.21 (0.95 -1.54)
Smoking behavioral dependence scale (11 items)			
Mild	1.0 (Ref)	1.0 (Ref)	1.0 (Ref)
Moderate	0.79 (0.06-10.38)	0.29 (0.03-3.01)	0.29 (0.03-2.80)
Strong to very strong	0.63 (0.05-8.20)	0.08 (0.01-0.94)*	0.11 (0.01-1.12)^
Readiness to Quit (1-10 scale, 1-8 apply to current smokers)	2.0 (1.18-3.38)*	1.73 (1.01-2.97)*	1.64 (1.01-2.66)*
50% Smoking reduction obtained at week 3	8.56 (1.53-47.96)*	44.0 (5.36-361.10)**	8.25 (1.50-45.43*)
ECPD: Week 1	1.15 (0.43-3.10)	1.54 (0.58-4.14)	1.35 (0.52-3.49)
ECPD: Week 3	1.96 (0.59-6.52)	2.62 (0.78-8.82)	2.82 (0.86-9.19)

*p<.05. **p<.01

Supplementary Table S3. Demographic associated factors for trajectory group assignment in multinomial logistic regression model with relative risk ratio (reference: maintainers)

	Relative Risk Ratio (95% CI)		
	**Rapid Reducers**	**Moderate Reducers**	**Slow Reducers**
Demographics			
Age in years	1.01 (0.843 - 1.222)	0.94 (0.765 - 1.160)	0.98 (0.819 - 1.181)
Gender			
Male (ref)	1.0 (Ref)	1.0 (Ref)	1.0 (Ref)
Female	1.18 (0.310 - 4.457)	8.78** (1.419 - 54.328)	2.51 (0.641 - 9.827)
Education			
High school or less (ref)	1.0 (Ref)	1.0 (Ref)	1.0 (Ref)
Some college	0.53 (0.082 - 3.459)	1.57 (0.210 - 11.815)	0.41 (0.062 - 2.728)
College/Post-Graduate	0.27 (0.034 - 2.097)	0.38 (0.039 - 3.696)	0.37 (0.051 - 2.723)
Race/Ethnicity			
Non-Hispanic African American/Black (Ref)	0.38 (0.061 - 2.342)	2.15 (0.266 - 17.427)	0.42 (0.063 - 2.818)
Non-Hispanic white	1.0 (Ref)	1.0 (Ref)	1.0 (Ref)
Other non-Hispanic	1.34 (0.090 - 19.734)	6.8 (0.401 - 115.136)	5.61 (0.498 - 63.169)
Hispanic of any race	0.48 (0.078 - 2.993)	1.85 (0.233 - 14.708)	0.81 (0.135 - 4.893)
Treatment			
Control (Placebo)	1.0 (Ref)	1.0 (Ref)	1.0 (Ref)
Active Nicotine	1.51 (0.377 - 6.076)	0.9 (0.197 - 4.077)	1.46 (0.369 - 5.791)

**p<.05

Supplementary Table S4. Demographic-associated factors for trajectory group assignment in multinomial logistic regression model with relative risk ratio (reference: maintainers [group 4])

	Relative Risk Ratio (95% CI)		
	**Rapid Reducers**	**Moderate Reducers**	**Slow Reducers**
Baseline Cigarettes Per Day			
< 15	1.0 (Ref)	1.0 (Ref)	1.0 (Ref)
15+	0.38 (0.043 - 3.385)	0.37 (0.038 - 3.665)	0.14* (0.015 - 1.292)
Heaviness of Smoking Index (0-5 scale)	0.8 (0.355 - 1.826)	0.93 (0.379 - 2.304)	1.25 (0.535 - 2.938)
Made serious quit attempts (>1 day) in last year	2.77 (0.492 - 15.613)	0.99 (0.150 - 6.575)	0.73 (0.121 - 4.363)
Confidence in quit ability	1.2 (0.838 - 1.720)	1.16 (0.770 - 1.747)	1.05 (0.737 - 1.495)
Smoking behavioral dependence scale			
Mild	1.0 (Ref)	1.0 (Ref)	1.0 (Ref)
Moderate	0.89 (0.051 - 15.573)	0.33 (0.020 - 5.476)	0.27 (0.019 - 3.624)
Strong to very strong	1.04 (0.052 - 21.046)	0.12 (0.006 - 2.675)	0.14 (0.008 - 2.494)
Readiness to Quit (1-10 scale, 1-8 apply to current smokers)	1.47 (0.698 - 3.080)	1.05 (0.486 - 2.287)	1.05 (0.506 - 2.168)
50% Smoking reduction obtained at week 3	3.37 (0.470 - 24.084)	27.52** (2.727 - 277.613)	5.93* (0.780 - 45.033)
ECPD: Week 1	0.92 (0.155 - 5.460)	1.18 (0.216 - 6.428)	1.56 (0.450 - 5.405)
ECPD: Week 3	2.13 (0.297 - 15.316)	1.67 (0.246 - 11.384)	2.08 (0.337 - 12.872)

*p<.05, **p<.01

Supplementary Table S5. Multinomial logistic estimates of classification into CC reduction

trajectory classes (Reference = Maintainers)

	Relative Risk Ratio (95% CI)		
	**Rapid Reducers**	**Moderate Reducers**	**Slow Reducers**
Readiness to Quit Smoking	1.96 (1.144-3.370)*	1.68 (0.974-2.899)	1.56 (0.960-2.541)
Heaviness of Smoking Index (0-5 scale)	0.63 (0.341 - 1.159)	0.74 (0.394 - 1.380)	1.01 (0.563 - 1.813)
Randomization to Nicotine Treatment	0.76 (0.187 - 3.101)	0.57 (0.134 - 2.465)	1.10 (0.292 - 4.172)

*p<.05

Supplementary Figure S2. Changes in Cigarette Cravings over Study Period by Trajectory Group

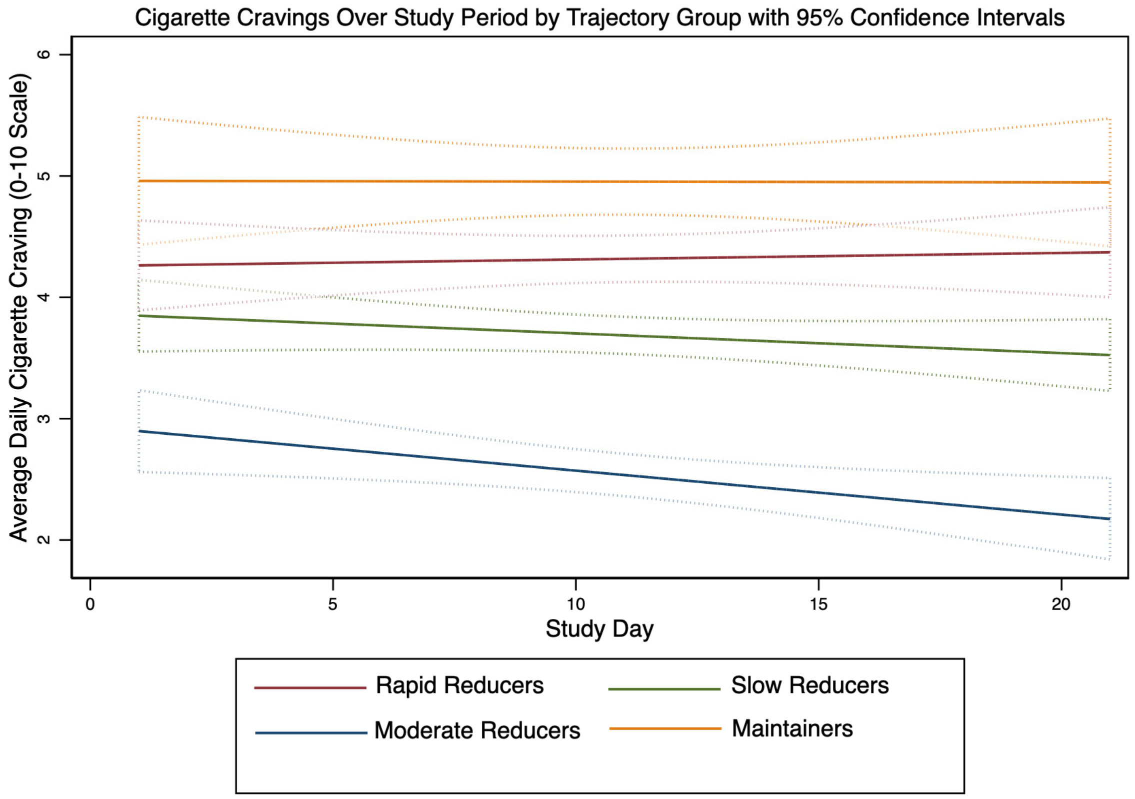

Supplementary Figure S3. Changes in Cigarette Cravings over Study Period by Trajectory Group

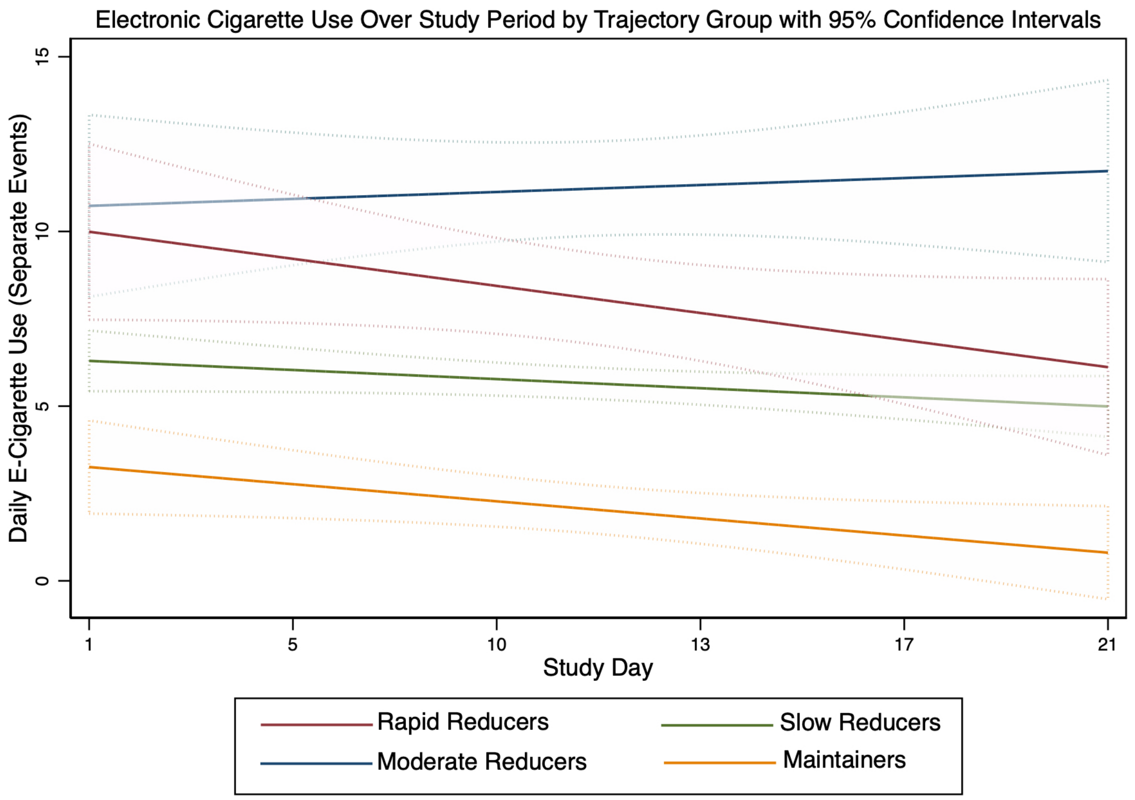

Supplement: Supplementary file 1 [file ijerph-19-07452-s001.zip › ijerph-1688564-supplementary.pdf]
